# Supplementary material for: EGFRvIII Confers Sensitivity to Saracatinib in a STAT5-Dependent Manner in Glioblastoma
Source: Int J Mol Sci. 2024 Jun 6;25(11):6279. doi: 10.3390/ijms25116279 (PMC11172708; doi:10.3390/ijms25116279)
Supplement: Supplementary file 1 [file ijms-25-06279-s001.zip › ijms-3019325 - supplementary.pptx]

## Slide 1
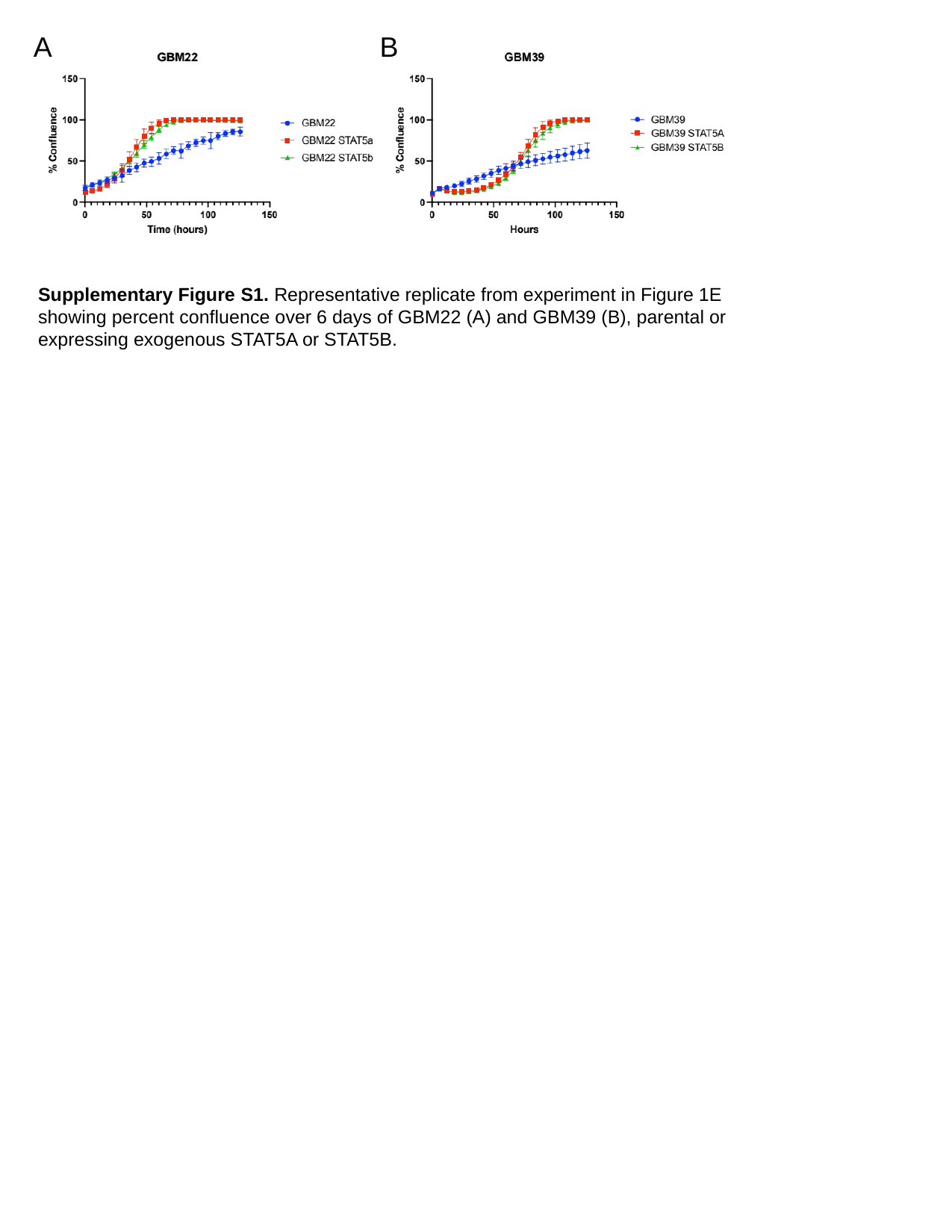

A
B
Supplementary Figure S1. Representative replicate from experiment in Figure 1E showing percent confluence over 6 days of GBM22 (A) and GBM39 (B), parental or expressing exogenous STAT5A or STAT5B.

## Slide 2
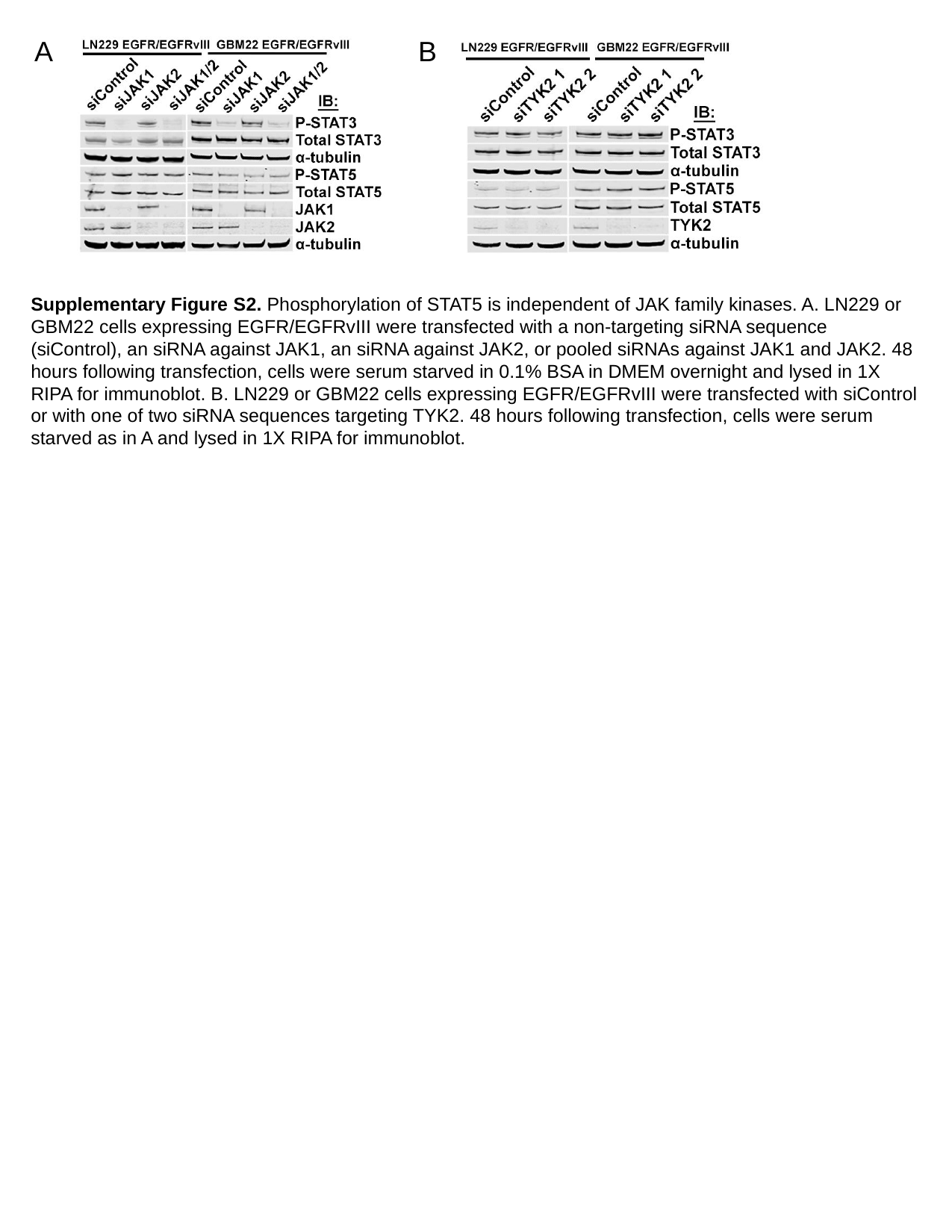

A
B
Supplementary Figure S2. Phosphorylation of STAT5 is independent of JAK family kinases. A. LN229 or GBM22 cells expressing EGFR/EGFRvIII were transfected with a non-targeting siRNA sequence (siControl), an siRNA against JAK1, an siRNA against JAK2, or pooled siRNAs against JAK1 and JAK2. 48 hours following transfection, cells were serum starved in 0.1% BSA in DMEM overnight and lysed in 1X RIPA for immunoblot. B. LN229 or GBM22 cells expressing EGFR/EGFRvIII were transfected with siControl or with one of two siRNA sequences targeting TYK2. 48 hours following transfection, cells were serum starved as in A and lysed in 1X RIPA for immunoblot.

## Slide 3
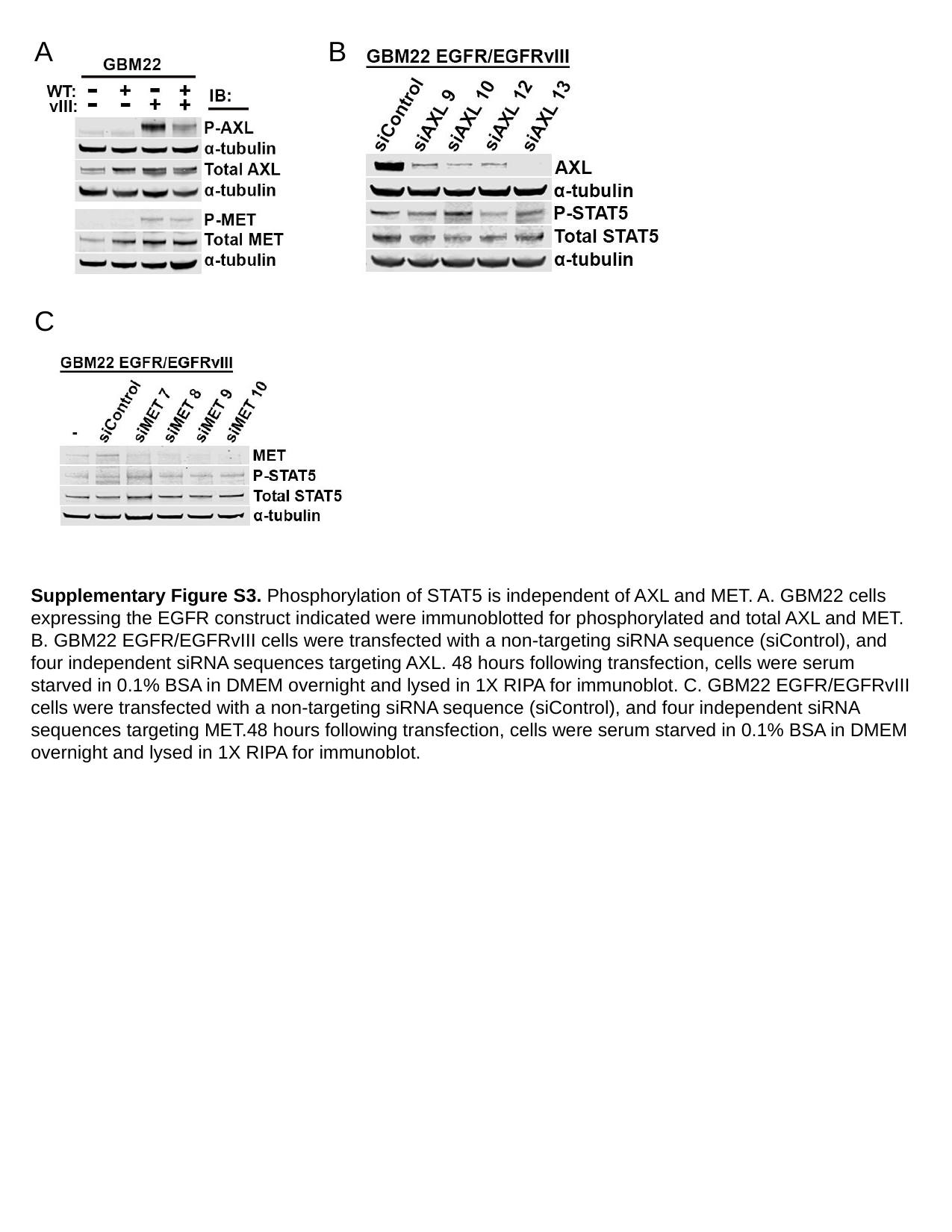

A
B
C
Supplementary Figure S3. Phosphorylation of STAT5 is independent of AXL and MET. A. GBM22 cells expressing the EGFR construct indicated were immunoblotted for phosphorylated and total AXL and MET. B. GBM22 EGFR/EGFRvIII cells were transfected with a non-targeting siRNA sequence (siControl), and four independent siRNA sequences targeting AXL. 48 hours following transfection, cells were serum starved in 0.1% BSA in DMEM overnight and lysed in 1X RIPA for immunoblot. C. GBM22 EGFR/EGFRvIII cells were transfected with a non-targeting siRNA sequence (siControl), and four independent siRNA sequences targeting MET.48 hours following transfection, cells were serum starved in 0.1% BSA in DMEM overnight and lysed in 1X RIPA for immunoblot.

## Slide 4
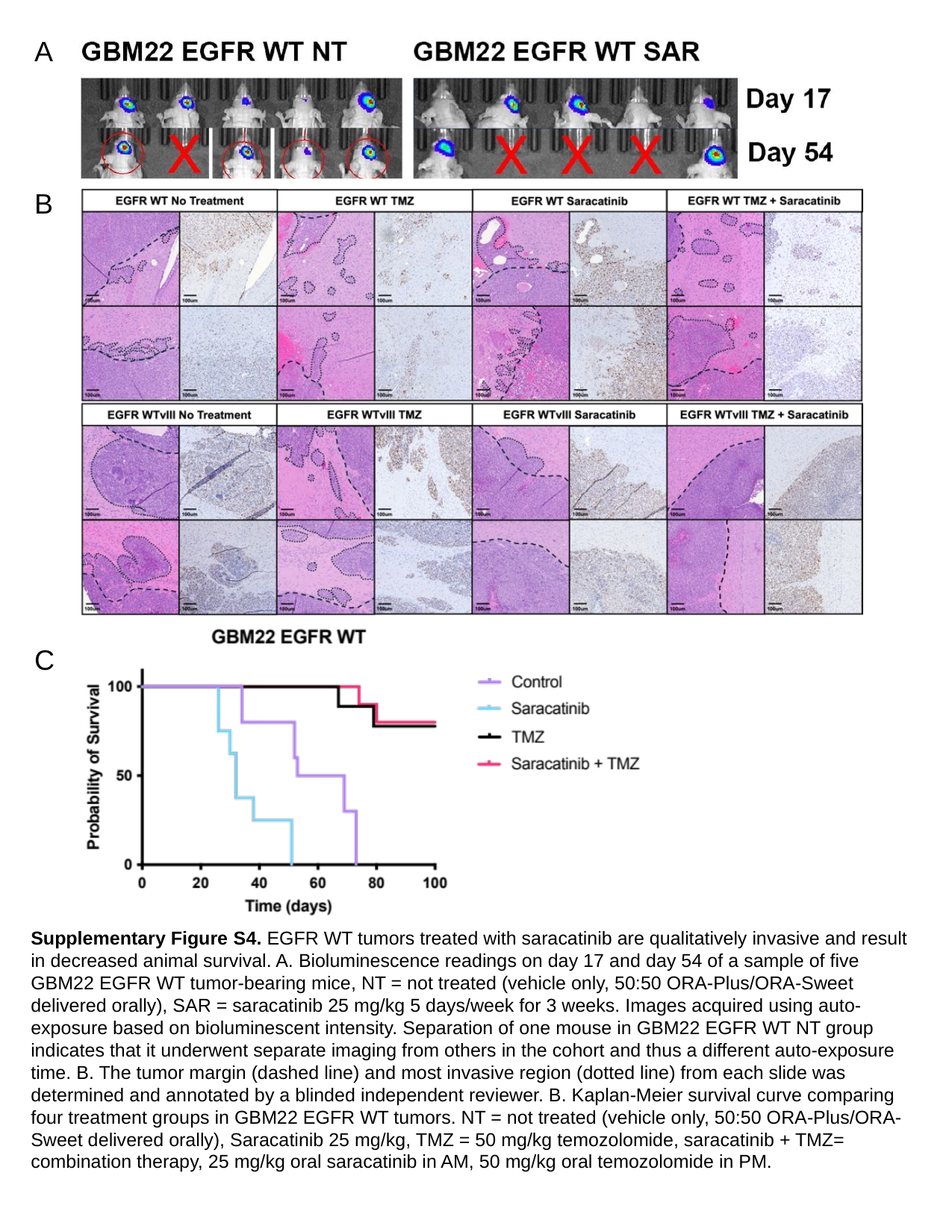

A
B
C
Supplementary Figure S4. EGFR WT tumors treated with saracatinib are qualitatively invasive and result in decreased animal survival. A. Bioluminescence readings on day 17 and day 54 of a sample of five GBM22 EGFR WT tumor-bearing mice, NT = not treated (vehicle only, 50:50 ORA-Plus/ORA-Sweet delivered orally), SAR = saracatinib 25 mg/kg 5 days/week for 3 weeks. Images acquired using auto-exposure based on bioluminescent intensity. Separation of one mouse in GBM22 EGFR WT NT group indicates that it underwent separate imaging from others in the cohort and thus a different auto-exposure time. B. The tumor margin (dashed line) and most invasive region (dotted line) from each slide was determined and annotated by a blinded independent reviewer. B. Kaplan-Meier survival curve comparing four treatment groups in GBM22 EGFR WT tumors. NT = not treated (vehicle only, 50:50 ORA-Plus/ORA-Sweet delivered orally), Saracatinib 25 mg/kg, TMZ = 50 mg/kg temozolomide, saracatinib + TMZ= combination therapy, 25 mg/kg oral saracatinib in AM, 50 mg/kg oral temozolomide in PM.
